# Old Colony Dental Society

**Published:** 1872-02

**Authors:** 


### Editorial.

### OLD COLONY DENTAL SOCIETY.

We recently had the pleasure of attending a meeting of this Society, held at North Bridgewater, Mass. The meeting was well attended, and its proceedings of an animated and interesting character.

In the discussions, many new thoughts and suggestions were elicited, pertaining to both principles and practice, illustrating the fact, as in all similar meetings we have attended, that men can not come together and compare practices and views, and discuss subjects, without gaining light and knowledge. Indeed, this is the great means of schooling and training those who are now in the profession; and it ought to be far more extensively and more earnestly employed than it is. Even though but half a dozen earnest men associate together, with a desire for improvement, they will accomplish much that could not otherwise be attained.

In association, union and harmony there is strength. It is true that here and there isolated individuals may seem to be strong, but it will usually be found to be a semblance instead of a reality.

Let every locality in which six to ten denti-ts may be gathered have its Dental Society, sustained regularly, and with a determination on the part of its members to do all they can for their mutual improvement and the elevation of the dental profession.

The Old Colony Dental Society occupies a territory of perhaps fifty miles square, and they meet every three months, at such place as they may appoint.

